# Impact on health outcomes and productive work of adding anti-PD-1 agents to treat early-stage cancers in the United Kingdom: a modelling study

**DOI:** 10.3389/fphar.2025.1613120

**Published:** 2025-10-30

**Authors:** Hannah Burton, Yan Zhi Tan, Sam Greenall, Catarina Neves, Neil Macdonald, Raquel Aguiar-Ibáñez

**Affiliations:** ^1^ MSD (UK) Ltd., London, United Kingdom; ^2^ Lumanity, Utrecht, Netherlands; ^3^ Lumanity, London, United Kingdom; ^4^ Merck Canada Inc., Kirkland, QC, Canada

**Keywords:** immunotherapy, immuno-oncology therapy, anti-PD-1, oncology, early-stage cancer, resource planning, productivity

## Abstract

**Background:**

Anti-PD-1 agents are recommended in adjuvant or perioperative settings in some early-stage cancers. The health and productivity benefits of anti-PD-1 use on a population level, however, are unknown.

**Methods:**

A decision model was developed to quantify the health and productivity outcomes of adding anti-PD-1 agents to traditional management strategies in the adjuvant or perioperative setting for melanoma stage IIB/IIC/III, triple negative breast cancer, and renal cell carcinoma in the United Kingdom. The model consisted of four separate Markov models and compared outcomes in two scenarios: one where anti-PD-1 agents are restricted to advanced/metastatic disease, against one where anti-PD-1 agents are used as adjuvant or perioperative therapy for early-stage cancers. Population and incidence inputs were obtained from nation-specific sources, while efficacy and quality of life data were informed by the individual trials. Productivity outcomes were estimated using a human capital approach.

**Results:**

Between 2023 and 2032, 57,075 (60.4%) of 94,426 patients with early-stage cancers eligible for adjuvant or perioperative treatment are estimated to receive anti-PD-1 agents. This was associated with an increase in total life years (8,878, 2.4%), quality-adjusted life-years (9,029, 3.1%), and event-/disease-/recurrence-free life years (25,149, 9.0%), and a reduction in progression events or recurrences (6,839, 16.8%), active metastatic treatments (4,845, 14.0%), and deaths (3,013, 16.2%). The clinical benefits also resulted in a gain in productive years (20,717, 17.6%).

**Conclusion:**

The use of anti-PD-1 agents in adjuvant or perioperative settings can lead to substantial health and productivity gains. Effective planning and investment are needed for timely access to these agents for patients.

## 1 Introduction

Cancer is a prevalent disease globally with severe health and economic implications. Like similar trends observed worldwide, the incidence of cancer in the UK has risen steadily over the past decades (Shelton et al., 2024; Bray et al., 2024); nearly half of the UK’s population is estimated to be diagnosed with cancer in their lifetime (Cancer Research UK, 2024; Aggarwal et al., 2024), and this incidence is set to rise further (Aggarwal et al., 2024).

Patients living with cancer face considerable health challenges and implications, especially in advanced disease. Despite improving survival rates due to increased implementation of cancer prevention and detection strategies, and advent of more effective cancer therapies (Shelton et al., 2024), cancer patients continue to experience impairments in daily activities, including patients with early-stage cancers (van Leeuwen et al., 2018; Roberts et al., 2024). Additionally, treatment and management of cancer is costly and resource intensive (Landeiro et al., 2024). This also leads to productivity losses for both patients and caregivers due to absences from work or requirements for informal care (Aguiar-Ibáñez et al., 2024). The wider economic impact of cancer on society is therefore extremely large, and was estimated at £18.9 billion in 2018 in England alone (Landeiro et al., 2024).

There has been a concerted effort to address this public health challenge in the United Kingdom. This has culminated in detailed strategies and long-term plans put forward by each of the four devolved nations (Scottish Government, 2023; Wales Cancer Network, 2023; Department of Health, 2022; Department of Health and Social Care, 2024). All nations emphasized the importance of earlier cancer diagnosis where prognosis is better, and disease burden lower, compared with cancers diagnosed at a late-stage. Wide-ranging measures, such as encouraging cancer screening uptake and coverage, optimizing diagnostic and referral pathways, and capacity building, were further identified to achieve this objective (Scottish Government, 2023; Wales Cancer Network, 2023; Department of Health, 2022; Department of Health and Social Care, 2024).

However, for health outcomes to be improved, earlier cancer diagnosis must be accompanied by timely receipt of effective treatments. While surgery remains one of the primary treatment modalities for many early-stage cancers, patients may still experience high risk of disease recurrence (Loibl et al., 2024; Michielin et al., 2019; Powles et al., 2024). Adjuvant or perioperative therapies that are given for a short period either after and/or before surgery have been shown to further reduce risk of recurrences and improve long-term outcomes. One example of such therapies includes the anti-PD-1 agents. These agents function by modulating immune checkpoints, thereby enhancing the immune system’s capacity to recognize and target malignant cells (LaFleur et al., 2018). Initially approved for advanced stage cancers, more recently anti-PD-1 agents have been shown to significantly improve event-free (EF), disease-free (DF) and recurrence-free (RF) survival when given as adjuvant or perioperative therapy for approximately 1 year in several early-stage cancers (Choueiri et al., 2024; Eggermont et al., 2021; Luke et al., 2022; Schmid et al., 2024; Wakelee et al., 2023). Furthermore, improvements in overall survival were also observed (Choueiri et al., 2024; Schmid et al., 2024; Wakelee et al., 2023).

However, these outcomes were reported in individual indications, and the potential health and productivity benefits of anti-PD-1 agents use across multiple early-stage cancer indications on a population level remain unknown. We therefore conducted this study to estimate the impact of wide-scale adoption of anti-PD-1 agents at a population level when used as adjuvant or perioperative treatment for some early-stage cancers in the United Kingdom.

## 2 Methods

### 2.1 Model overview

**FIGURE 1:**
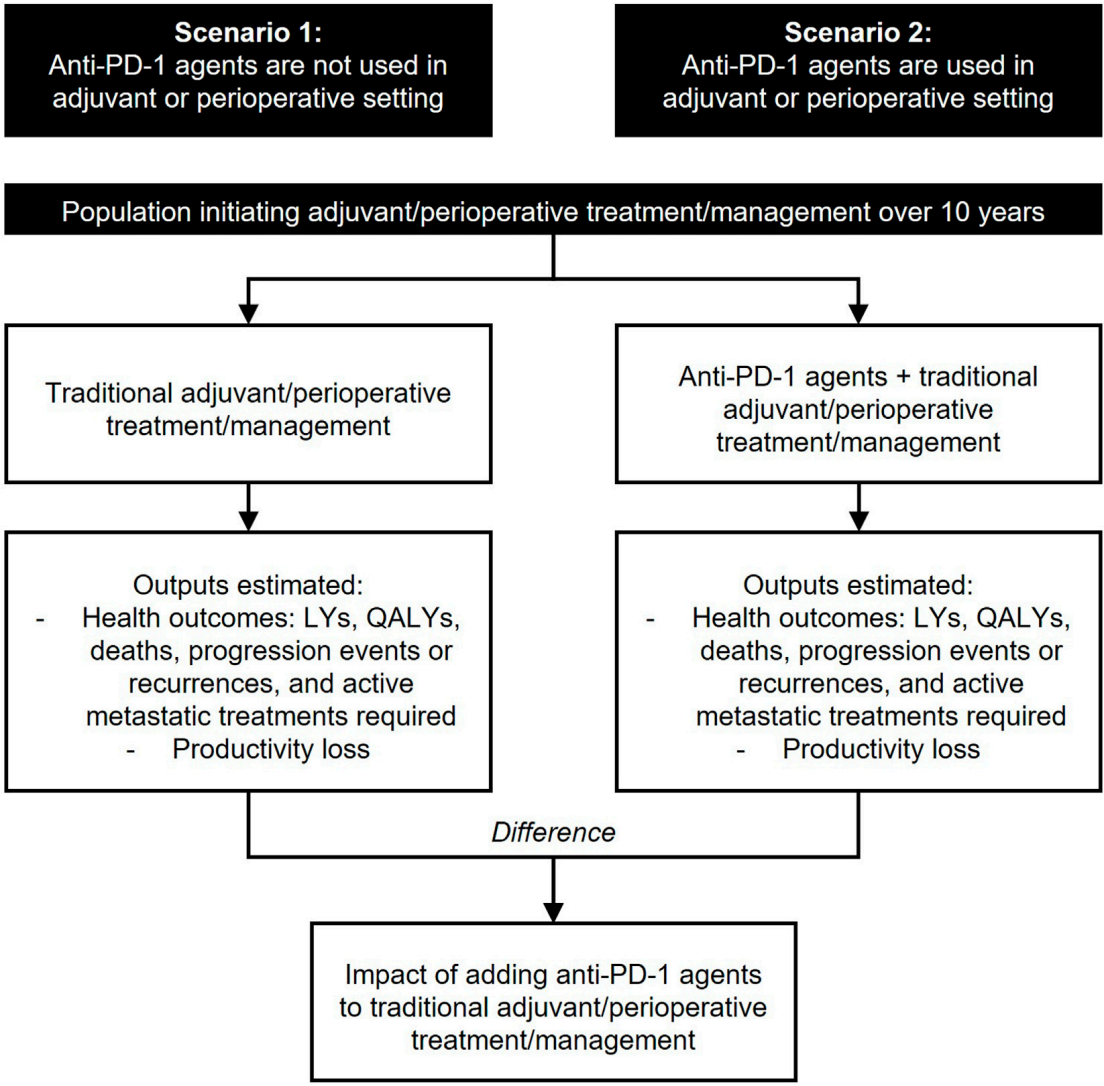
Analysis overview. Key: LY, life-year, QALY, quality-adjusted life-year.

A decision model was developed to quantify the health and productivity outcomes of adding anti-PD-1 agents to traditional management strategies in the adjuvant or perioperative setting for three early-stage cancers over a 10-year period in the UK from 2023 to 2032. This time horizon was selected to balance capturing the potential long-term benefits of anti-PD-1 agents with the greater uncertainty in outcomes when extrapolating further into the future and unknown future changes in the external environment. The model examined and compared the outcomes in two scenarios – one where anti-PD-1 agents are not used as adjuvant or perioperative therapy for early-stage cancers (Scenario 1), against one where anti-PD-1 agents are used as adjuvant or perioperative therapy for early-stage cancers (Scenario 2) (Figure 1). Three licensed indications (melanoma stage IIB/IIC/III, triple negative breast cancer [TNBC] and renal cell carcinoma [RCC]) where anti-PD-1 agents had been approved by the Medicines & Healthcare products Regulatory Agency (MHRA) and recommended, based on clinical and cost-effectiveness, by the National Institute for Health and Care Excellence (NICE) and the Scottish Medicines Consortium (SMC) for routine use throughout the UK were selected (National Institute for Health and Care Excellence, 2022a; National Institute for Health and Care Excellence, 2022b; National Institute for Health and Care Excellence, 2022c; National Institute for Health and Care Excellence, 2022d; Scottish Medicines Consortium, 2023a; Scottish Medicines Consortium, 2023b; Scottish Medicines Consortium, 2019; Scottish Medicines Consortium, 2022a). To ensure methodological robustness, the decision model comprised of four separate Markov models (melanoma stage IIB/C, melanoma stage III, TNBC, and RCC) that had been submitted to, and accepted by, health technology assessment (HTA) bodies (National Institute for Health and Care Excellence, 2022a; National Institute for Health and Care Excellence, 2022b; National Institute for Health and Care Excellence, 2022c; National Institute for Health and Care Excellence, 2022d; Scottish Medicines Consortium, 2023a; Scottish Medicines Consortium, 2023b; Scottish Medicines Consortium, 2019; Scottish Medicines Consortium, 2022a). All outcomes were discounted at 3.5% following prevailing NICE guidance (National Institute for Health and Care Excellence, 2022e).

Analyses were conducted separately for each devolved UK nation (i.e., England, Scotland, Wales, and Northern Ireland) and then aggregated to obtain results for the entire United Kingdom. All models shared the same structure with four mutually exclusive health states. New incident cohorts entered the model in the EF/DF/RF state on a weekly basis, initiated treatment with an anti-PD-1 agent or conventional strategies in the adjuvant or perioperative setting (as per the relevant license), and were subjected to a risk of three competing events: locoregional recurrence (LR), distant metastases (DM), or death, the absorbing state (Supplementary Figure S1). To enable granular calculation of health state occupancy and outcomes, a 1-week cycle length was used.

### 2.2 Model inputs

#### 2.2.1 Population

All starting patient characteristics in the model were consistent with the population from relevant trials at baseline. Eligible population size for each indication was derived by multiplying the general population size with indication- and nation-specific incidence and eligibility rates (Supplementary Figure S2; Supplementary Table S1). General population size was retrieved from the Office for National Statistics (ONS) for all nations (Office for National Statistics, 2024a). The estimated annual growth rate for each nation was also derived from ONS and assumed to be equal across the 10-year horizon (Office for National Statistics, 2024b). Incidence rates for each of the three tumour types were derived from country specific databases (National Disease Registration Service, 2024; Northern Ireland Cancer Registry, 2024a; Northern Ireland Cancer Registry, 2024b; Northern Ireland Cancer Registry, 2024c; Public Health Scotland, 2021; Public Health Scotland, 2024a; Public Health Scotl and, 2024; Public Health Scotland, 2024b; Public Health Wales, 2024); 2022 rates were used where possible as these were the most recent data available, although values for Wales were for 2021 as 2022 data were not available at the time of the analysis.

#### 2.2.2 Treatment mix

Treatment mix in the adjuvant or perioperative setting in Scenarios 1 and 2 was informed by published resource impact templates (RITs) that reflected NHS England predictions of treatment practices and uptake of anti-PD-1 agents at the time of the NICE technology appraisal guidance (Table 1) (National Institute for Health and Care Excellence, 2021a; National Institute for Health and Care Excellence, 2022f; National Institute for Health and Care Excellence, 2022g; National Institute for He alth and Care Excellence, 2022). The same mix was assumed across the model time horizon.

**TABLE 1:** Treatment mix in the adjuvant or perioperative setting, by indication.

	Melanoma, stage IIB/C^a^	Melanoma, stage III^a^ ^,^ ^b^	RCC^c^	TNBC^d^
Scenario 1: Anti-PD-1 agents are not used as adjuvant or perioperative therapy in early-stage cancers				
Anti-PD-1 agents	0.0%	0.0%	0.0%	0.0%
Dabrafenib + trametinib	NA	39.8%*^e^	NA	NA
Chemotherapy	NA	NA	NA	100.0%
Watchful waiting	100.0%	60.2%*	100.0%	NA
Scenario 2: Anti-PD-1 agents are used as adjuvant or perioperative therapy in early-stage cancers				
Anti-PD-1 agents	50.0%	59.1%	65.0%	65.0%
Dabrafenib + trametinib	NA	30.2%	NA	NA
Chemotherapy	NA	NA	NA	35.0%
Watchful waiting	50.0%	10.7%	35.0%	NA
Sensitivity analysis: 100% of anti-PD-1 use in Scenario 2				
Anti-PD-1 agents	100.0%	100.0%	100.0%	100.0%
Dabrafenib + trametinib	NA	0.0%	NA	NA
Chemotherapy	NA	NA	NA	0.0%
Watchful waiting	0.0%	0.0%	00.0%	NA

*Proportion of patients receiving dabrafenib + trametinib in Scenario 1 was estimated by assuming that 80% of patients with a BRAF mutation from KEYNOTE-054 receive active treatment. The remaining patients were then assumed to undergo watchful waiting.

References:

^a^ NICE, TA837 Resource Impact Report (National Institute for Health and Care Excellence, 2022f).

^b^ NICE, TA684 Resource Impact Report (National Institute for Health and Care Excellence, 2022).

^c^ NICE, TA830 Resource Impact Template (National Institute for He alth and Care Excellence, 2022).

^d^ NICE, TA851 Resource Impact Report (National Institute for Health and Care Excellence, 2022g).

^e^ Eggermont et al. (2021).

Key: NA, not applicable; RCC, renal cell carcinoma; TNBC, triple negative breast cancer.

Upon entry into the DM state (from EF/DF/RF or LR states), patients were assumed to initiate first-line metastatic treatment contingent on: (a) previous treatments used in the adjuvant or perioperative setting; and (b) whether re-treatment with anti-PD-1 agents was allowed. The choice of possible therapies was based on treatment options approved by NICE and the SMC, and the proportion of patients receiving each therapy was informed by market research and expert clinical opinion. Re-treatment with anti-PD-1 agents in the metastatic setting was permitted for patients who entered the DM state at least 6 months after completing adjuvant or perioperative therapy.

#### 2.2.3 Efficacy

Transition probabilities from the EF/DF/RF states were derived from parametric modelling fitted to patient-level data from the respective trials of pembrolizumab (Eggermont et al., 2021; Long et al., 2022; Powles et al., 2022; Schmid et al., 2022). Extrapolations of trial data were aligned with those used in the corresponding HTA cost-effectiveness models, where the parametric distributions were selected based on a combination of statistical fit, visual fit to observed trial data, and clinical plausibility (National Institute for Health and Care Excellence, 2022a; National Institute for Health and Care Excellence, 2022b; National Institute for Health and Care Excellence, 2022c; National Institute for Health and Care Excellence, 2022d; Scottish Medicines Consortium, 2023a; Scottish Medicines Consortium, 2023b; Scottish Medicines Consortium, 2019; Scottish Medicines Consortium, 2022a). Long-term survival estimates were validated against relevant external sources of data where available. Transitions from the LR state were estimated from the relevant trials, or real-world evidence if trial data were insufficient.

For dabrafenib + trametinib in melanoma stage III, transitions from the RF state were estimated by applying a hazard ratio against the RFS curve of pembrolizumab, while transitions from the LR state were assumed to be similar to that of pembrolizumab. For simplicity, efficacy of other anti-PD-1 agents (applicable only for melanoma stage III) was assumed to be equivalent to that of pembrolizumab (Franken et al., 2019). The efficacy of individual first-line subsequent treatments was used to derive transitions from the DM state to death. All transitions to death were adjusted for background mortality.

#### 2.2.4 Utilities

Utility values were assigned to each health state to represent the differences in health-related quality of life (HRQoL) between different recurrence statuses. All health-state utility values (HSUVs) were mapped to EQ-5D-3L using the UK value set based on the EQ-5D-5L questionnaire responses collected in the pivotal trials (van Hout et al., 2012; Dolan, 1997), or other published sources where necessary, and adjusted for age and sex (Ara and Brazier, 2010). Adverse event (AE) disutilities associated with Grade 3+ adverse events that occurred in ≥5% of patients in either arm of the respective clinical trials were applied once at treatment initiation to capture the impact of treatment tolerability on HRQoL.

#### 2.2.5 Productivity losses

Acknowledging that there may be wider societal benefits associated with improving health outcomes, patients’ and caregivers’ productivity was examined in the model using a human capital approach. Standard working hours per week were assumed to be 36.6 for all nations, based on UK labour estimates (Office for National Statistics, 2024c). Inputs relating to the impact of early stage cancer on patients’ and caregivers’ presenteeism and absenteeism within these standard working hours were obtained from a survey on the human and economic burden of early-stage cancer and applied across all indications (Supplementary Table S2) (Aguiar-Ibáñez et al., 2024).

### 2.3 Outcomes

The modelled outcomes for Scenarios 1 and 2 included total and event-/disease-/recurrence-free life years and quality-adjusted life-years (QALYs), progression events or recurrences, number of active metastatic treatments, deaths, and productivity losses. The impact of adding anti-PD-1 agents to the early-stage setting was estimated by taking the difference between the outcomes of the two scenarios. For each outcome, model results for the four individual indications were then summed to derive the total impact and appropriately reflect the incidence of each cancer.

To examine the potential benefits of broader adoption of anti-PD-1 agents beyond current estimates, a sensitivity analysis was conducted by assuming all patients will be treated with anti-PD-1 agents (i.e. 100% uptake). While a small proportion of patients may be contraindicated to immuno-oncology (IO) therapy, this analysis will represent the most optimistic impact should there be full uptake of anti-PD-1 agents.

## 3 Results

**FIGURE 2:**
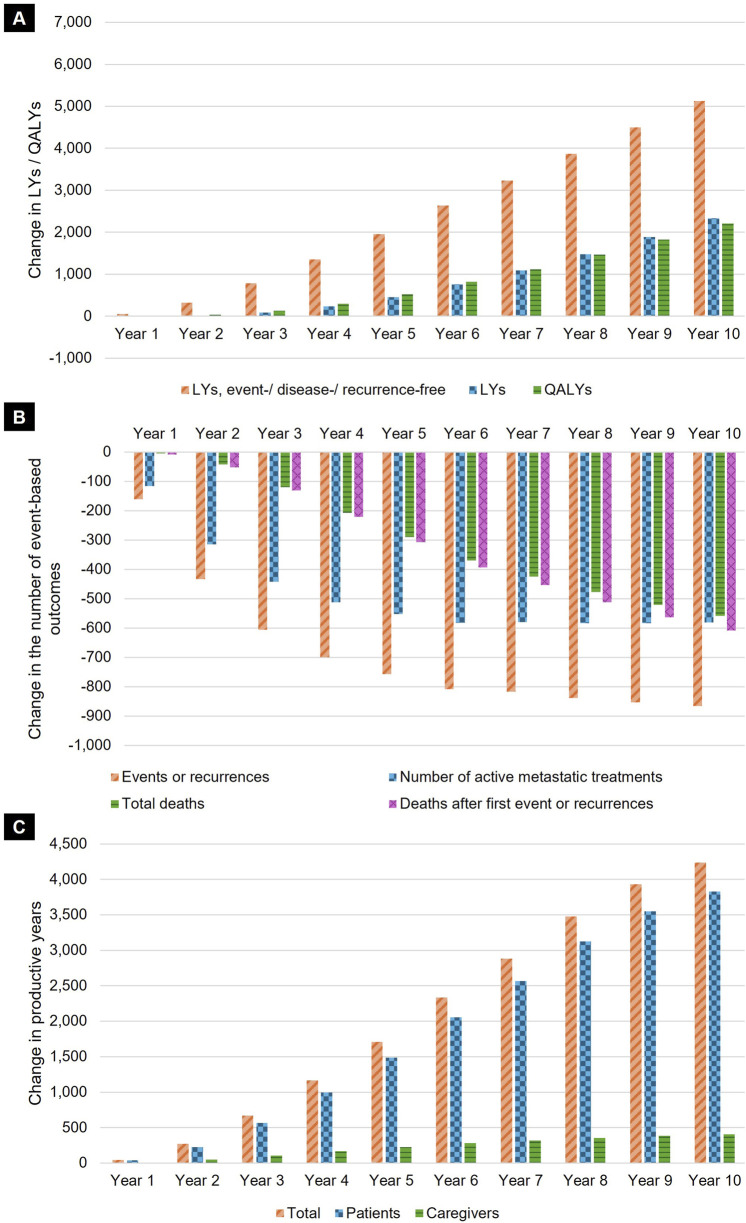
Annual impact of using anti-PD-1 agents in early-stage cancer. Results presented reflect the annual impact of the modelled uptake of anti-PD-1 agents (i.e., Scenario 2 vs. Scenario 1) on the total estimated **(A)** LYs, event-/disease-/recurrence-free LYs, and QALYs; **(B)** event-based outcomes (progression events or recurrences, deaths, deaths after first event or recurrence, and number of active metastatic treatments after progression or recurrence); and **(C)** productive years gained (increase in productive working time for patients and their caregivers, as a result of improved health outcomes). Key: LY, life-year; QALY, quality-adjusted life-year.

Between 2023 and 2032, 57,075 (60.4%) of 94,426 patients with early-stage cancers eligible for adjuvant or perioperative treatment are estimated to receive adjuvant or perioperative anti-PD-1 agents for the treatment of melanoma stage IIB/C and III, RCC, and TNBC across all four devolved nations (Supplementary Table S3). Using anti-PD-1 agents in early-stage cancer was estimated to result in overall health gains with increases in total life years (8,878, 2.4%), QALYs (9,029, 3.1%), and event-/disease-/recurrence-free life years (25,149, 9.0%) (Table 2). The use of anti-PD-1 agents was also associated with reductions in progression events or recurrences (6,839, 16.8%), active metastatic treatments (4,845, 14.0%), and deaths (3,013, 16.2%). The gains in total life years, event-/disease-/recurrence-free life years and QALYs were also expected to accumulate steadily over the 10-year horizon, reflecting long-term clinical benefit resulting from reductions in progression events or recurrences, and increasing number of patients treated over the years (Figure 2). Benefits of treatment were seen across the four cancer types (Supplementary Table S4).

**TABLE 2:** Estimated outcomes, by nation.

	Life years, event-/disease-/recurrence-free	Life years, total	QALYs	Progression events or recurrences	Number of active metastatic treatments	Deaths	Productive years lost*
Scenario 1: Anti-PD-1 agents are not used as adjuvant or perioperative therapy in early-stage cancers							
Total	278,778	362,670	295,852	40,698	34,644	18,632	117,596
England	234,494	305,151	248,949	34,222	29,066	15,632	98,841
Scotland	23,255	30,145	24,608	3,345	2,896	1,565	9,764
Wales	14,287	18,648	15,193	2,159	1,859	986	6,171
Northern Ireland	6,742	8,726	7,102	972	824	450	2,820
Scenario 2: Anti-PD-1 agents are used as adjuvant or perioperative therapy in early-stage cancers							
Total	303,927	371,549	304,882	33,859	29,800	15,619	96,878
England	255,638	312,603	256,532	28,473	25,016	13,102	81,429
Scotland	25,358	30,888	25,357	2,778	2,488	1,312	8,033
Wales	15,583	19,114	15,671	1,802	1,590	829	5,099
Northern Ireland	7,348	8,944	7,322	806	706	376	2,318
Absolute difference (%)							
Total	25,149 (9.0%)	8,878 (2.4%)	9,029 (3.1%)	−6,839 (−16.8%)	−4,845 (−14.0%)	−3,013 (−16.2%)	−20,717 (−17.6%)
England	21,143 (9.0%)	7,452 (2.4%)	7,582 (3.0%)	−5,748 (−16.8%)	−4,050 (−13.9%)	−2,529 (−16.2%)	−17,413 (−17.6%)
Scotland	2,103 (9.0%)	743 (2.5%)	749 (3.0%)	−566 (−16.9%)	−407 (−14.1%)	−253 (−16.2%)	−1,731 (−17.7%)
Wales	1,296 (9.1%)	466 (2.5%)	478 (3.1%)	−358 (−16.6%)	−269 (−14.5%)	−156 (−15.9%)	−1,072 (−17.4%)
Northern Ireland	606 (9.0%)	217 (2.5%)	221 (3.1%)	−167 (−17.1%)	−118 (−14.4%)	−74 (−16.4%)	−502 (−17.8%)

*Since the outcome reported is ‘productive years lost’, a decrease in productive years lost when Scenario 2 is compared to Scenario 1 (i.e., a negative value) is to be interpreted as productive years gained. For example, the Total −20, 717 productive years lost indicates a total of +20,717 productive years gained, derived from 96,878 productive years lost in Scenario 2 minus 117,596 productive years lost in Scenario 1.

Key: QALY, quality-adjusted life year.

As a result of the reductions in progression events, recurrences and death, use of anti-PD-1 agents was also associated with overall productivity gains due to lower presenteeism and absenteeism for both patients and caregivers (Figure 2). This is estimated to result in a gain of 20,717 productive years (17.6%) across the entire 10-year horizon.

Increasing anti-PD-1 agent uptake in the adjuvant or perioperative setting to all eligible patients resulted in further health and productivity gains (Supplementary Table S5). Assuming 100% uptake of anti-PD-1 agents can lead to further gains of 58.6%, 60.7%, and 60.6% in life years, event-/disease-/recurrence-free life years, and QALYs, respectively, compared with the primary analysis. Additionally, there were greater reductions in progression events or recurrences, number of active metastatic treatments, and deaths (60.3%, 71.0%, and 62.6% respectively), corresponding to larger productivity gains (12,598 productive years, 60.8%).

## 4 Discussion

Previous studies have demonstrated the clinical efficacy and cost-effectiveness of anti-PD-1 agents as treatments for early-stage cancers. Building on this, our study demonstrates that substantial health and productivity gains can be realized on a population level with adjuvant or perioperative anti-PD-1 use across multiple early-stage cancers throughout the United Kingdom. These benefits are considerable even with modest predictions regarding the extent of anti-PD-1 uptake in each indication and are estimated to be even greater with higher levels of uptake. Furthermore, the potential reduction in cancer progression events or recurrences and number of active metastatic treatments is pronounced across all nations. While differences in magnitude were observed across individual cancer types, primarily reflecting variations in relative efficacy, place in therapy, and disease incidence, the overall health and societal benefits associated with the introduction of anti-PD-1 agents were consistently demonstrated. In addition to direct impact on a patient’s life expectancy and quality of life, these reductions may avert potential costs and resource use associated with later lines of treatment for metastatic disease as well as terminal care, both of which are often costly and require extensive healthcare resource use (Ray et al., 2013). This study estimated productivity gains of 17.6%, suggesting that adjuvant or perioperative anti-PD-1 agent use in early cancers may lead to notable societal benefits.

Our findings underscore the importance of advancing access for new treatment options, such as anti-PD-1 agents, in early-stage cancers. The health and productivity benefits that increased uptake of such agents may confer are synergistic to the intended outcomes of enhanced screening, detection, and diagnosis outlined by all four devolved nations in their cancer strategies. Specifically, all nations have committed to increasing the share of early-stage cancer diagnosis. Under the NHS Long Term Plan, NHS England has committed to increase the proportion of cancers diagnosed at stages I and II in England from half to three-quarters of cases by 2028 (National Health Services, 2019). This is anticipated to result in 55,000 more people being expected to survive their cancer for at least 5 years annually. Similarly, the Scottish government plans to reduce the proportion of late-stage cancer diagnoses from 42% to 24% by 2033 (Scottish Government, 2023). While no specific targets have been set by Northern Ireland and Wales, plans to enhance early-stage cancer diagnosis were regarded as important measures to tackle growing health inequalities in both nations (Wales Cancer Network, 2023; Department of Health, 2022).

However, the benefits of early diagnosis can only be achieved when coupled with timely initiation of effective treatments. Cancers that are diagnosed early are more likely to undergo surgical treatment with a curative intent (Cancer Research UK, 2024). Despite this, the chance of recurrence following surgery may still be high in aggressive cancers such as TNBC and melanoma (Romano et al., 2010; Costa and Gradishar, 2017). Current clinical evidence supports the addition of adjuvant or perioperative treatments, such as anti-PD-1 agents, to reduce the risk of relapse and mortality after surgical resection compared to traditional adjuvant or perioperative options such as chemotherapy or watchful waiting (Choueiri et al., 2024; Eggermont et al., 2021; Luke et al., 2022; Schmid et al., 2024). Economic analyses have frequently demonstrated that these new treatments are also cost-effective, and have resulted in recommendations by NICE and the SMC for nationwide routine commissioning (National Institute for Health and Care Excellence, 2022a; National Institute for Health and Care Excellence, 2022b; National Institute for Health and Care Excellence, 2022c; National Institute for Health and Care Excellence, 2022d; Scottish Medicines Consortium, 2023a; Scottish Medicines Consortium, 2023b; Scottish Medicines Consortium, 2019; Scottish Medicines Consortium, 2022a; National Institute for Health and Care Excellence, 2021b; Scottish Medicines Consortium, 2018). Facilitating and expanding consistent access to these agents will therefore be needed to translate the advantages of early diagnosis into more substantial health gains, and to ensure equitable access across the United Kingdom.

Despite the importance of prompt initiation of cancer treatment, waiting times to start treatment are currently long and disparate across all four devolved nations. The proportion of patients meeting the 62-day standard, a measure where patients begin treatment within 62 days of an urgent referral, ranged from 38% in Northern Ireland to 72% in Scotland in the fourth quarter of 2022, well below the targets for each nation (Scottish Government, 2023; Wales Cancer Network, 2023; Department of Health, 2022; Office for National Statistics, 2025). An international benchmarking study further highlights that UK cancer patients face longer waits and lower treatment uptake than those in comparable countries (McPhail et al., 2024). Treatment for cancer is often time-sensitive, and delays in initiating treatment may reduce patients’ capacity to benefit. In some instances, patients may even miss the opportunity to receive treatment if the disease progresses. Effective treatment options, and the corresponding prognosis, may also be limited for advanced disease, and treatment delays have been associated with poorer outcomes across many cancers (Hanna et al., 2020). This may result in poorer health outcomes on a population level that may lead to wider societal impact.

Understanding potential reasons for delays in diagnosis and subsequently starting cancer treatment may provide better insights into how earlier diagnosis and access to effective treatments can be improved. First, ongoing capacity and workforce challenges in healthcare systems across all four nations have been cited as key reasons for delayed access to cancer services, including consultations and drug administrations (Wales Cancer Network, 2023; Department of Health, 2022; Department of Health and Social Care, 2024). Access to general practitioners and emergency departments has also been impaired, resulting in delayed or missed diagnoses and, by extension, treatment initiation (Department of Health and Social Care, 2024). Despite concerns that increased adoption of immunotherapies may exacerbate the already strained healthcare system (Scottish Government, 2023; Wales Cancer Network, 2023), encouraging its use in early-stage cancer represents a partial shift in capacity allocation rather than isolated increased demand for healthcare resources. Investing in optimal care for patients with early-stage cancers, which includes the use of adjuvant and perioperative treatments such as anti-PD-1 agents, may contribute to alleviation of capacity requirements in the advanced setting in the longer-term. Results may be even more pronounced with larger and wider adoption of these therapies, as demonstrated in this study.

Secondly, delivery of systemic anti-cancer therapy (SACT), including anti-PD-1 agents, has traditionally been confined to hospitals which may deter some patients from initiating treatment due to cost or inconvenience (Scottish Government, 2023; Department of Health, 2022). Channelling newly diagnosed patients into an already constrained system may lead to further delay in initiation of cancer care. Novel methods of treatment delivery could be explored to ease hospital footfall; suggestions to shift some SACT delivery to homes and community settings may increase accessibility and appeal (Franken et al., 2020), particularly for those who are initiating cancer treatment.

Finally, the role of geographic and socioeconomic factors should also be considered. Ensuring equitable access is a stated priority in each nation’s cancer plan (Scottish Government, 2023; Department of Health, 2022; National Health Services, 2019; Welsh Government, 2025). However, people living in more rural or more deprived areas of the UK are less likely to have their cancer diagnosed at an early stage and are more likely to have longer waits to begin cancer treatment (Cancer Research UK, 2025; Dobson et al., 2022). Whilst the reasons for these disparities are multifaceted and complex, targeted interventions within these areas to improve symptom awareness, health literacy and screening attendance, to remove or reduce practical barriers to accessing care, and to ensure healthcare providers are adequately resourced would support earlier diagnoses and shorter intervals to initiation of effective treatments overall.

### 4.1 Strengths and limitations

This study is the first to examine the impact of the widespread adoption of anti-PD-1 agents for multiple early-stage cancers in the UK, using models accepted by HTA agencies. Outcomes in this model were accrued from the point of adjuvant and perioperative treatment initiation with new incident cohorts entering the model on a weekly basis until the end of the study time horizon; this provides a dynamic and more accurate representation of overall benefits compared to typical models where outcomes are modelled from a single treatment initiation timepoint. In addition, incidence and population projections specific to each of the four UK nations were used wherever possible to increase significance of the results. Further, subsequent treatments were comprehensively modelled, which enhanced the relevance of the outcomes to real-world clinical practice. Whilst this study focused on the impact in the UK, the overall conclusions are also applicable to other countries and healthcare systems.

Our study had some limitations. First, the 10-year study horizon may not be sufficient to confirm the long-term trajectory of current findings, but is intended to balance capturing potential benefits with future uncertainty and is also aligned with the time frame considered in the government’s 10-Year Health Plan for England (Department of Health and Social Care, 2025). Second, productivity inputs were proxied from a survey of US patients given the lack of relevant UK-specific values (Aguiar-Ibáñez et al., 2024), although overall conclusions relating to societal benefits observed in this analysis would not be expected to change. Third, uptake of anti-PD-1 agents was assumed to be constant across the time horizon. Thus any changes to the uptake due to newly approved agents in the future could not be accounted for. Similarly, constant incidence rates were applied; if cancer incidence rates continue to rise over time, the benefits of anti-PD-1 agents estimated here may be slightly underestimated. Fourth, while the model used the latest trial data-cuts available at the time of the HTA appraisals, more mature data can be used to validate current results in the future. Further, in the absence of real-world evidence, this analysis is based on clinical trial data; trial populations tend to be fitter than real-world populations, therefore it is plausible that the benefits of anti-PD-1 agents in the real-world differ slightly to those estimated in this study. Lastly, while this study focused on three specific indications driven by availability of patient-level data, other anti-PD-1 agents have also been approved by NICE and the SMC for routine use in further tumour types (Scottish Medicines Consortium, 2018; National Institute for Health and Care Excellence, 2021c; National Institute for Health and Care Excellence, 2022h; National Institute for Health and Care Excellence, 2023; Scottish Medicines Consortium, 2022b; Scottish Medicines Consortium, 2023c), and the number of recommended early-stage indications is set to grow as the evidence base evolves. Therefore, the full extent of the health and societal impact of anti-PD-1 agents reported in this study is likely to be underestimated and the results of this study are likely to be conservative.

## 5 Conclusion

Access to anti-PD-1 agents as adjuvant or perioperative therapy for early-stage cancers is estimated to deliver substantial health benefits at a UK population level over a 10-year time horizon. These benefits are expected to rise in the longer-term, following trends observed from this study, and will increase further if uptake exceeds predictions. Adopting anti-PD-1 agents in the treatment of early-stage cancers may also lead to substantial productivity gains. However, these gains may not be fully realized if ongoing access challenges, particularly capacity constraints within the health system, are not addressed. It is therefore imperative to effectively plan and invest in the necessary resources and implementation strategies to ensure consistent access and timely initiation of appropriate treatment for patients with early-stage cancer across the whole of the United Kingdom.

## Data Availability

The original contributions presented in the study are included in the article/Supplementary Material, further inquiries can be directed to the corresponding author.
