# Supplementary material for: Impact on health outcomes and productive work of adding anti-PD-1 agents to treat early-stage cancers in the United Kingdom: a modelling study
Source: Front Pharmacol. 2025 Oct 30;16:1613120. doi: 10.3389/fphar.2025.1613120 (PMC12612631; doi:10.3389/fphar.2025.1613120)
Supplement: Supplementary file 1 [file DataSheet1.pdf]

# *Supplementary Material*

**Figure A1: Model structure ........................................ 2**

**Figure A2: Patient flow for each indication ........................................ 3**

**Table A1: Population and epidemiology inputs for each indication, by nation ........................................ 5**

**Table A2: Proportion of patients and caregivers experiencing presenteeism and absenteeism ... 6**

**Table A3: Estimated number of patients treated with adjuvant or perioperative anti-PD-1 agents by cancer type from 2023 to 2032 ........................................ 7**

**Table A4: Estimated outcomes, by cancer ........................................ 8**

**Table A5: Sensitivity analysis (100% uptake of anti-PD-1 agents) – estimated outcomes, by nation ........................................ 8**

# 1 Supplementary Tables and Figures

## Figure A1: Model structure

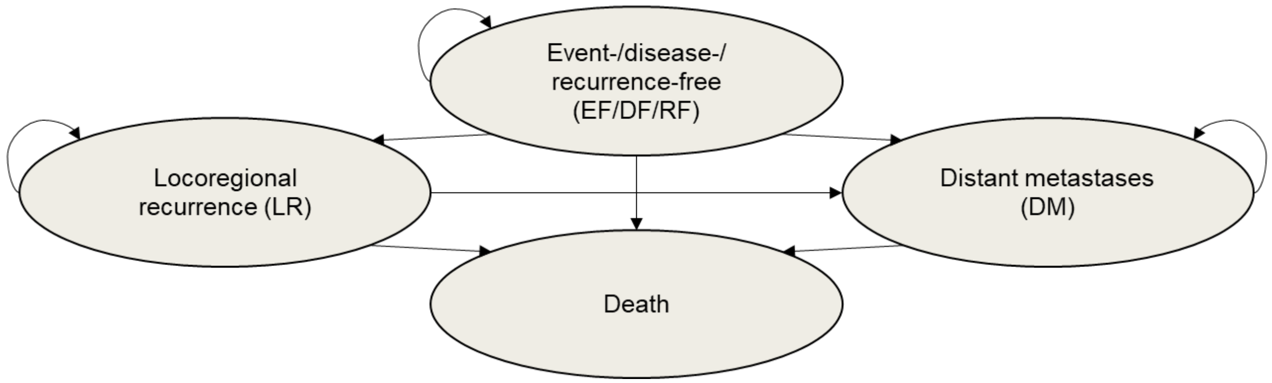

**Figure A2: Patient flow for each indication**

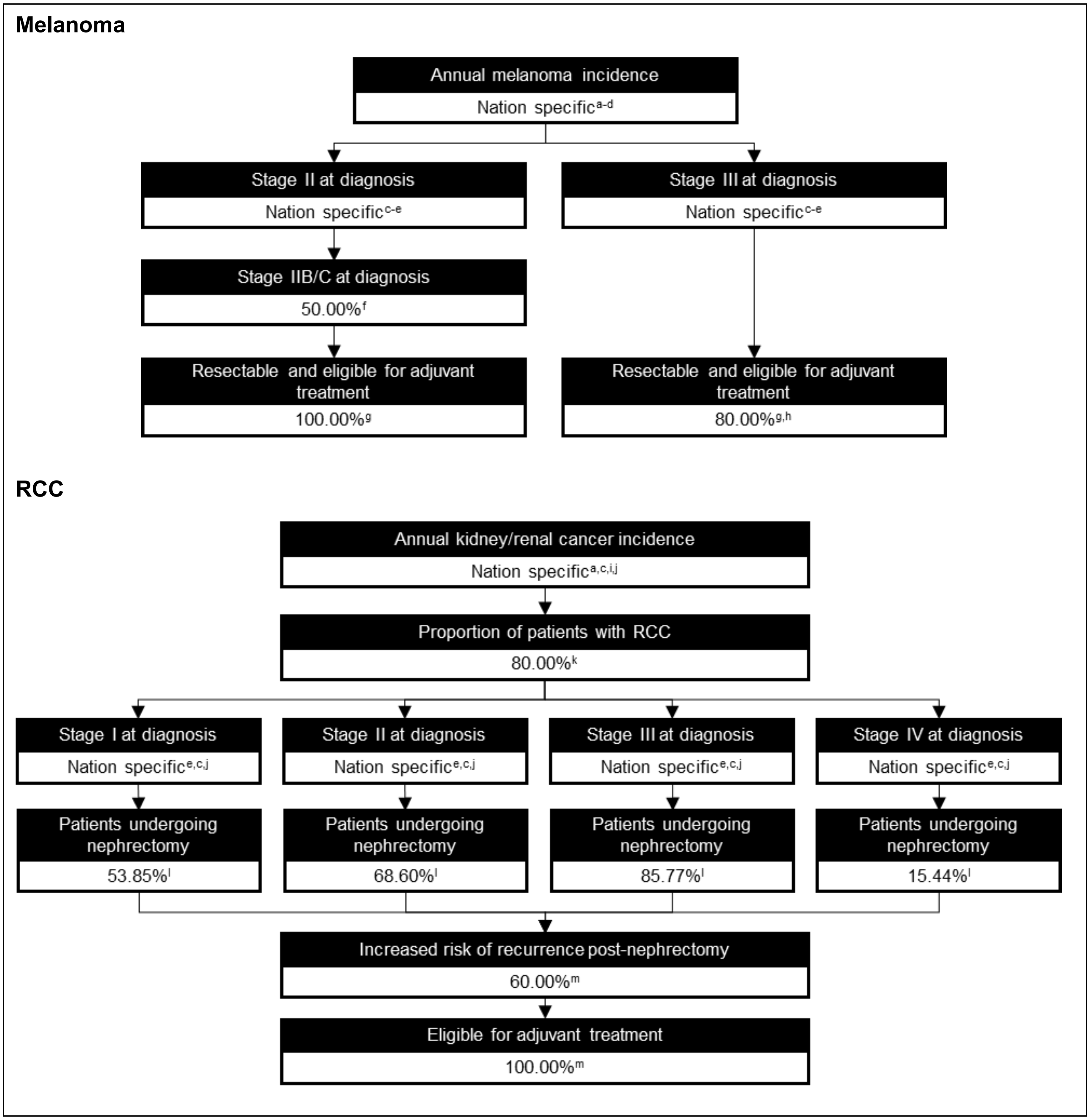

**TNBC**

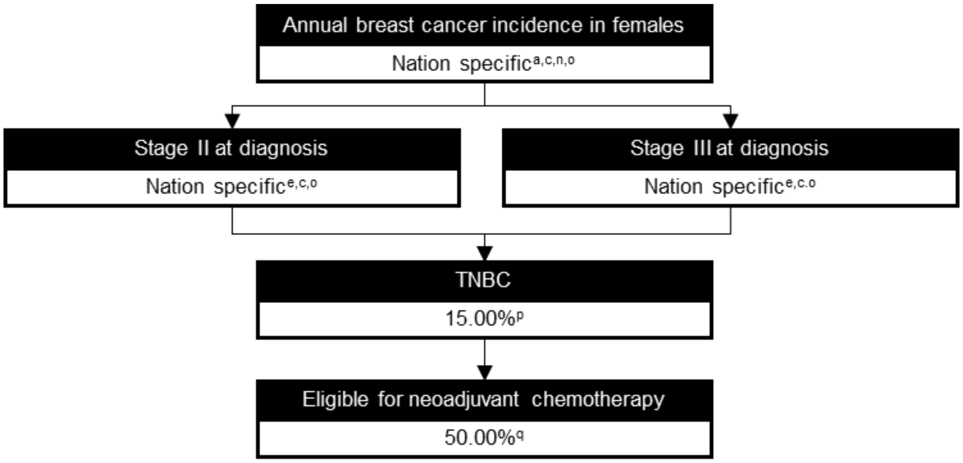


**Note**: For simplicity, the model estimated the eligible population based on stage at diagnosis – patients who may become eligible after recurrence from an earlier stage were not included.
**Key**: RCC, renal cell carcinoma; TNBC, triple negative breast cancer.
**References**: [a]National Disease Registration Service (1) [b]Public Health Scotland (2) [c]Public Health Wales (3) [d]Northern Ireland Cancer Registry (4) [e]National Disease Registration Service (5) [f]Poklepovic and Luke 2020 (6) [g]NICE TA837 Resource Impact Report (7); [h]NICE TA684 Resource Impact Report (8); [i]Public Health Scotland (9) [j]Northern Ireland Cancer Registry (10) [k]Cancer Research UK (11) [l]National Disease Registration Service (12) [m]NICE TA830 Resource Impact Report (13) [n]Public Health Scotland (14) [o]Northern Ireland Cancer Registry (15) [p]Cancer Research UK (16) [q]NICE TA851 Resource Impact Report (17)

**Table A1: Population and epidemiology inputs for each indication, by nation**

| Population input | | England | Scotland* | Wales† | Northern Ireland |
|---|---|---|---|---|---|
| Population size | | 57,690,323 (18) | 5,490,100 (18) | 3,164,404 (18) | 1,920,382 (18) |
| Population growth | | 0.68% (19) | 0.32% (19) | 0.30%(19) | 0.47% (19) |
| Females | | 50.97% (18) | 51.39% (18) | 50.93% (18) | 50.77% (18) |
| Melanoma | Annual incidence | 31.0/100,000 (1) | 30.4/100,000 (2) | 32.8/100,000 (3) | 26.8/100,000 (4) |
| | Stage II at diagnosis | 20.62% (5) | 20.62% (5)* | 24.89% (3) | 18.70% (4) |
| | Stage III at diagnosis | 8.84% (5) | 8.84% (5)* | 11.69% (3) | 9.40% (4) |
| RCC | Annual incidence | 18.2/100,000 (1) | 20.5/100,000 (9) | 18.2/100,000 (3) | 14.8/100,000 (10) |
| | Stage I at diagnosis | 51.78% (5) | 51.78% (5) | 44.55% (3) | 54.80% (10) |
| | Stage II at diagnosis | 6.48% (5) | 6.48% (5)* | 7.39% (3) | 6.40% (10) |
| | Stage III at diagnosis | 20.44% (5) | 20.44% (5)* | 16.73% (3) | 20.20% (10) |
| | Stage IV at diagnosis | 21.30% (5) | 21.30% (5)* | 31.32% (3) | 18.70% (10) |
| TNBC | Annual incidence for breast cancer in females | 173.9/100.000 (1) | 183.5/100,000 (14) | 185.3100,000 (3) | 164.7/100,000 (15) |
| | Stage II at diagnosis | 40.66% (5) | 40.66% (5)* | 41.66% (3) | 40.00% (15) |
| | Stage III at diagnosis | 9.34% (5) | 9.34% (5)* | 11.46% (3) | 11.00% (15) |

**Note:** *Proportion of patients diagnosed at each cancer stage for Scotland was assumed to be the same as that of England as no Scottish-specific inputs were available; † Incidence and staging data for Wales are from 2021 as 2022 data were not available at the time of the analysis.
**Key:** RCC, renal cell carcinoma; TNBC, triple negative breast cancer
**References:** [1] Office for National Statistics, [2] Office for National Statistics, [3] National Disease Registration Service, [4] Public Health Scotland, [5] Public Health Wales, [6] Northern Ireland Cancer Registry, [7] National Disease Registration Service, (9) Public Health Scotland, (10) Northern Ireland Cancer Registry, (14) Public Health Scotland, (15) Northern Ireland Cancer Registry.

**Table A2: Proportion of patients and caregivers experiencing presenteeism and absenteeism**

| State | Patients | | | Caregivers | | | |
|---|---|---|---|---|---|---|---|
| | Presenteeism | | Absenteeism | Patients requiring carer support | Presenteeism | | Absenteeism |
| | Patients experiencing work impairment | Amount of work impairment | Patients absent from work | | Carers experiencing work impairment | Amount of work impairment | Carers absent from work |
| Recurrence-free | 0% | 0% | 0% | 0% | 0% | 0% | 0% |
| Locoregional | 94% | 17% | 15% | 44% | 95% | 9% | 8% |
| Metastatic | 88% | 53% | 29% | 44% | 86% | 12% | 28% |

**Note:** All proportions were derived from a survey on employment, finances, and productivity for early-stage cancer patients and caregivers in the US by Aguiar-Ibáñez et al. (20)

**Table A3: Estimated number of patients treated with adjuvant or perioperative anti-PD-1 agents by cancer type from 2023 to 2032**

| | Melanoma Stage II | Melanoma Stage III | RCC | TNBC |
|---|---|---|---|---|
| **Total** | 11,270 | 9,224 | 21,256 | 15,326 |
| England | 9,496 | 7,701 | 17,993 | 12,854 |
| Scotland | 873 | 708 | 1,893 | 1,280 |
| Wales | 655 | 581 | 879 | 784 |
| Northern Ireland | 246 | 234 | 490 | 408 |

**Key:** RCC, renal cell carcinoma; TNBC, triple negative breast cancer

**Table A4: Estimated outcomes, by cancer type**

| | Life years, event-/ disease-/ recurrence-free | Life years, total | QALYs | Progression events or recurrences | Number of active metastatic treatments | Deaths | Productive years lost* |
|---|---|---|---|---|---|---|---|
| **Scenario 1: Anti-PD-1 agents are not used as adjuvant or perioperative therapy in early-stage cancers** | | | | | | | |
| Total | 278,778 | 362,670 | 295,852 | 40,698 | 34,644 | 18,632 | 117,596 |
| Melanoma IIB/C | 64,712 | 88,256 | 73,523 | 11,249 | 10,022 | 4,150 | 27,090 |
| Melanoma III | 36,811 | 54,381 | 44,167 | 9,951 | 9,729 | 4,797 | 28,942 |
| RCC | 93,630 | 127,386 | 106,491 | 12,816 | 10,435 | 5,845 | 42,552 |
| TNBC | 83,625 | 92,648 | 71,671 | 6,682 | 4,458 | 3,840 | 19,011 |
| **Scenario 2: Anti-PD-1 agents are used as adjuvant or perioperative therapy in early-stage cancers** | | | | | | | |
| Total | 303,927 | 371,549 | 304,882 | 33,859 | 29,800 | 15,619 | 96,878 |
| Melanoma IIB/C | 68,978 | 89,384 | 74,886 | 10,138 | 8,924 | 3,776 | 23,916 |
| Melanoma III | 42,884 | 56,915 | 46,831 | 8,159 | 7,914 | 4,005 | 23,618 |
| RCC | 103,876 | 130,467 | 109,457 | 10,555 | 9,660 | 4,766 | 34,420 |
| TNBC | 88,190 | 94,784 | 73,708 | 5,007 | 3,301 | 3,072 | 14,923 |
| **Absolute difference (%)** | | | | | | | |
| Total | 25,149 (9.0%) | 8,878 (2.4%) | 9,029 (3.1%) | -6,839 (-16.8%) | -4,845 (-14.0%) | -3,013 (-16.2%) | -20,717 (-17.6%) |
| Melanoma IIB/C | 4,266 (6.6%) | 1,128 (1.3%) | 1,363 (1.9%) | -1,111 (-9.9%) | -1,098 (-11.0%) | -374 (-9.0%) | -3,174 (-11.7%) |
| Melanoma III | 6,072 (16.5%) | 2,534 (4.7%) | 2,664 (6.0%) | -1,792 (-18.0%) | -1,815 (-18.7%) | -792 (-16.5%) | -5,324 (-18.4%) |
| RCC | 10,246 (10.9%) | 3,080 (2.4%) | 2,966 (2.8%) | -2,261 (-17.6%) | -775 (-7.4%) | -1,079 (-18.5%) | -8,132 (-19.1%) |
| TNBC | 4,565 (5.5%) | 2,136 (2.3%) | 2,037 (2.8%) | -1,675 (-25.1%) | -1,157 (-26.0%) | -768 (-20.0%) | -4,088 (-21.5%) |

**Key:** QALY, quality-adjusted life year; RCC, renal cell carcinoma; TNBC, triple negative breast cancer.

**Table A5: Sensitivity analysis (100% uptake of anti-PD-1 agents) – estimated outcomes, by nation**

| | Life years, event-/ disease-/ recurrence-free | Life years, total | QALYs | Progression events or recurrences | Number of active metastatic treatments | Deaths | Productive years lost |
|---|---|---|---|---|---|---|---|
| **Scenario 1: Anti-PD-1 agents are not used as adjuvant or perioperative therapy in early-stage cancers** | | | | | | | |
| Total | 278,778 | 362,670 | 295,852 | 40,698 | 34,644 | 18,632 | 117,596 |
| *England* | 234,494 | 305,151 | 248,949 | 34,222 | 29,066 | 15,632 | 98,841 |
| *Scotland* | 23,255 | 30,145 | 24,608 | 3,345 | 2,896 | 1,565 | 9,764 |
| *Wales* | 14,287 | 18,648 | 15,193 | 2,159 | 1,859 | 986 | 6,171 |
| *Northern Ireland* | 6,742 | 8,726 | 7,102 | 972 | 824 | 450 | 2,820 |
| **Scenario 2: Anti-PD-1 agents are used as adjuvant or perioperative therapy in early-stage cancers** | | | | | | | |
| Total | 319,188 | 376,748 | 310,349 | 29,737 | 26,360 | 13,733 | 84,280 |
| *England* | 268,470 | 316,968 | 261,122 | 25,007 | 22,139 | 11,519 | 70,840 |
| *Scotland* | 26,626 | 31,320 | 25,809 | 2,439 | 2,204 | 1,155 | 6,988 |
| *Wales* | 16,381 | 19,389 | 15,964 | 1,584 | 1,394 | 729 | 4,437 |
| *Northern Ireland* | 7,712 | 9,070 | 7,454 | 707 | 623 | 330 | 2,016 |
| **Absolute difference (%)** | | | | | | | |
| Total | 40,410 (14.5%) | 14,077 (3.9%) | 14,497 (4.9%) | -10,961 (-26.9%) | -8,284 (-23.9%) | -4,899 (-26.3%) | -33,315 (-28.3%) |
| *England* | 33,976 (14.5%) | 11,817 (3.9%) | 12,173 (4.9%) | -9,214 (-26.9%) | -6,926 (-23.8%) | -4,113 (-26.3%) | -28,002 (-28.3%) |
| *Scotland* | 3,371 (14.5%) | 1,176 (3.9%) | 1,201 (4.9%) | -905 (-27.1%) | -692 (-23.9%) | -410 (-26.2%) | -2,776 (-28.4%) |
| *Wales* | 2,094 (14.7%) | 741 (4.0%) | 770 (5.1%) | -575 (-26.7%) | -465 (-25.0%) | -256 (-26.0%) | -1,734 (-28.1%) |
| *Northern Ireland* | 970 (14.4%) | 343 (3.9%) | 353 (5.0%) | -266 (-27.3%) | -201 (-24.4%) | -119 (-26.6%) | -804 (-28.5%) |

| Absolute difference in sensitivity analysis (100% uptake of anti-PD-1 agents) results vs base case | | | | | | | |
|---|---|---|---|---|---|---|---|
| Total | 15,261 (60.7%) | 5,199 (58.6%) | 5,468 (60.6%) | -4,122 (60.3%) | -3,439 (71.0%) | -1,886 (62.6%) | -12,598 (60.8%) |
| *England* | 12,832 (60.7%) | 4,365 (58.6%) | 4,591 (60.5%) | -3,466 (60.3%) | -2,876 (71.0%) | -1,583 (62.6%) | -10,589 (60.8%) |
| *Scotland* | 1,267 (60.3%) | 433 (58.2%) | 452 (60.4%) | -339 (59.9%) | -285 (69.9%) | -157 (61.9%) | -1,045 (60.4%) |
| *Wales* | 798 (61.5%) | 275 (59.0%) | 293 (61.3%) | -218 (61.0%) | -195 (72.5%) | -100 (64.1%) | -662 (61.7%) |
| *Northern Ireland* | 363 (59.9%) | 126 (57.9%) | 132 (59.8%) | -99 (59.5%) | -83 (70.2%) | -46 (62.1%) | -302 (60.1%) |

**Key:** QALY, quality-adjusted life year

## 2 References


1. National Disease Registration Service. Cancer Incidence and Mortality, 2022 (2024) [23 September 2025]. Available from: https://nhsd-ndrs.shinyapps.io/incidence_and_mortality/.

2. Public Health Scotland. Cancer Incidence in Scotland - Skin, 2022 (2024) [23 September 2025]. Available from: https://publichealthscotland.scot/publications/cancer-incidence-in-scotland/cancer-incidence-in-scotland-to-december-2022/data-files/#Skin.

3. Public Health Wales. Welsh Cancer Intelligence and Surveillance Unit (Wcisu), 2021 (2024) [23 September 2025]. Available from: https://phw.nhs.wales/services-and-teams/welsh-cancer-intelligence-and-surveillance-unit-wcisu/.

4. Northern Ireland Cancer Registry. Malignant Melanoma, 2022 (2024) [23 September 2025]. Available from: https://www.qub.ac.uk/research-centres/nicr/CancerInformation/official-statistics/BySite/MalignantMelanoma/.

5. National Disease Registration Service. Stage Group by Sub-Integrated Care Board Location (Sub-Icbl), Integrated Care Board (Icb) or Cancer Alliance (Ca) by Cancer Type for 16 Cancer Types, 2022 (2024) [23 September 2025]. Available from: https://nhsd-ndrs.shinyapps.io/staging_data_in_england/.

6. Poklepovic AS, Luke JJ. Considering Adjuvant Therapy for Stage Ii Melanoma. *Cancer* (2020) 126(6):1166-74. Epub 20191223. doi: 10.1002/cncr.32585.

7. National Institute for Health and Care Excellence. Resource Impact Report: Pembrolizumab for Adjuvant Treatment of Resected Stage 2b or 2c Melanoma (Ta837) (2022) [28 October 2024]. Available from: https://www.nice.org.uk/guidance/ta837/resources/resource-impact-report-pdf-11256975613.

8. National Institute for Health and Care Excellence. Resource Impact Report: Nivolumab for Adjuvant Treatment of Completely Resected Melanoma with Lymph Node Involvement or Metastatic Disease (Ta684) (2021) [28 October 2024]. Available from: https://www.nice.org.uk/guidance/ta684/resources/resource-impact-report-pdf-9024957757.

9. Public Health Scotland. Cancer Incidence in Scotland - Urinary Tract, 2022 (2024) [23 September 2025]. Available from: https://publichealthscotland.scot/publications/cancer-incidence-in-scotland/cancer-incidence-in-scotland-to-december-2022/data-files/#Urinary%20tract.

10. Northern Ireland Cancer Registry. Kidney Cancer, 2022 (2024) [23 September 2025]. Available from: https://www.qub.ac.uk/research-centres/nicr/CancerInformation/official-statistics/BySite/Kidneycancer/.

11. Cancer Research UK. Types and Grades of Kidney Cancer (2024) [28 October 2024]. Available from: https://www.cancerresearchuk.org/about-cancer/kidney-cancer/stages-types-grades/types-grades.

12. National Disease Registration Service. Cancer Treatments 2013-2022, Kidney Cancer, 2022. (2024) [23 September 2025]. Available from: https://nhsd-ndrs.shinyapps.io/cancer_treatments/.

13. National Institute for Health and Care Excellence. Resource Impact Report: Pembrolizumab for Adjuvant Treatment of Renal Cell Carcinoma (Ta830) (2022) [28 October 2024]. Available from: https://www.nice.org.uk/guidance/ta830/resources/resource-impact-report-pdf-11253520477.

14. Public Health Scotland. Cancer Incidence in Scotland - Breast, 2022 (2024) [23 September 2025]. Available from: https://publichealthscotland.scot/publications/cancer-incidence-in-scotland/cancer-incidence-in-scotland-to-december-2022/data-files/#Breast.

15. Northern Ireland Cancer Registry. Breast Cancer and Insitu Tumours, 2022 (2024) [23 September 2025]. Available from: https://www.qub.ac.uk/research-centres/nicr/CancerInformation/official-statistics/BySite/Breastcancerandinsitutumours/.

16. Cancer Research UK. Triple Negative Breast Cancer (2024) [26 September 2024]. Available from: https://www.cancerresearchuk.org/about-cancer/breast-cancer/types/triple-negative-breast-cancer.

17. National Institute for Health and Care Excellence. Resource Impact Report: Pembrolizumab for Neoadjuvant and Adjuvant Treatment of Triple-Negative Early or Locally Advanced Breast Cancer (Ta851) (2022) [28 October 2024]. Available from: https://www.nice.org.uk/guidance/ta851/resources/resource-impact-report-pdf-11315579437.

18. Office for National Statistics. Estimates of the Population for the Uk, England, Wales, Scotland, and Northern Ireland (2024) [updated March 20243 December 2024]. Available from: https://www.ons.gov.uk/peoplepopulationandcommunity/populationandmigration/populationestimates/datasets/populationestimatesforukenglandandwalesscotlandandnorthernireland.

19. Office for National Statistics. Population Estimates Time Series Dataset (2024) [updated March 20243 December 2024]. Available from: https://www.ons.gov.uk/peoplepopulationandcommunity/populationandmigration/populationestimates/datasets/populationestimatestimeseriesdataset.

20. Aguiar-Ibáñez R, McQuarrie K, Jayade S, Penton H, DiGiovanni L, Raina R, et al. Impact of Recurrence on Employment, Finances, and Productivity for Early-Stage Cancer Patients and Caregivers: Us Survey. *Future Oncol* (2024):1-17. Epub 20241216. doi: 10.1080/14796694.2024.2436342.
